# Prevalence of Cervical Enamel Projections and Enamel Pearls in Saudi Adults: A Cone-Beam Computed Tomography Study

**DOI:** 10.3390/diagnostics16152455

**Published:** 2026-08-04

**Authors:** Hussam M. Alqahtani, Afrah M. Alossimi, Lama A. Alosail, Yasser N. Alotaibi

**Affiliations:** 1 Department of Preventive Dental Science, College of Dentistry, King Saud Bin Abdulaziz University for Health Sciences, Riyadh 11481, Saudi Arabia; 2 King Abdullah International Medical Research Center, Ministry of National Guard Health Affairs, Riyadh 11481, Saudi Arabia; alossimiafrah@gmail.com (A.M.A.); alosail007@gmail.com (L.A.A.); 3 Dental Services, Ministry of National Guard Health Affairs, Riyadh 11481, Saudi Arabia; 4 Department of Periodontics, King Abdulaziz Medical City, Ministry of National Guard-Health Affairs, Riyadh 11481, Saudi Arabia; 5 General Dentist, King Saud University, Riyadh 12372, Saudi Arabia; ynalotaibi@outlook.com

**Keywords:** cone-beam computed tomography, cervical enamel projection, enamel pearls, molar anatomy, prevalence, periodontology

## Abstract

**Background/Objectives**: To determine the prevalence and distribution of cervical enamel projections (CEPs) and enamel pearls (EPs) in Saudi adults using cone-beam computed tomography (CBCT). **Methods**: In this retrospective cross-sectional study, CBCT scans of 1376 consecutive patients attending postgraduate periodontics clinics between January 2024 and February 2025 were screened. After applying eligibility criteria, 153 patients (732 first and second molars) were included. Three calibrated examiners independently assessed each molar for CEPs and EPs. Inter-rater agreement was quantified using Fleiss’s kappa. Prevalence was reported at the patient and tooth levels with 95% confidence intervals, and bivariate associations with age, gender, and medical history were tested using chi-square, Fisher’s exact, and Mann–Whitney U tests. **Results**: Of 732 molars in 153 patients, the patient-level prevalence was 29.4% for CEPs and 3.3% for EPs; tooth-level prevalence was 7.7% and 0.7%, respectively. Grade I CEPs were the most common, and mandibular first molars were most affected. Inter-rater agreement was excellent (Fleiss’s κ = 0.86 for CEPs; κ = 1.00 for EPs). Neither dental anomaly was associated with age, gender, or medical history. **Conclusions**: CEPs are common in Saudi adults, while EPs are uncommon. Neither dental anomaly is associated with patient demographics or systemic health, supporting their developmental rather than acquired origin.

## 1. Introduction

Periodontal disease is a chronic, destructive inflammatory disease of the tissues supporting teeth that is caused by complex interactions between the immune system of the human body and pathogenic oral microbes [1]. This disease has a spectrum from reversible gingivitis to irreversible periodontitis, leading to attachment loss, mobility, and tooth loss [2]. Periodontitis is a leading cause of tooth loss in adults. The global prevalence of severe disease is estimated at 10–15% [3,4]. The prevalence of periodontal disease in Saudi Arabia is 51%, according to data from 14 studies involving 6596 people [5].

In molar teeth, the furcation area is particularly susceptible to attachment loss as a part of periodontitis. Its complex anatomy hinders both self-performed plaque control and professional debridement. Several local tooth-related anatomical factors also predispose molars to localized periodontal breakdown in this region [6,7]. Cervical enamel projections (CEPs) and enamel pearls are the most frequently described developmental anomalies of this type [8].

CEPs are continuous extensions of enamel from the cementoenamel junction (CEJ) towards the furcation [8]. Enamel surfaces support only epithelial attachments, not connective tissue attachment. Therefore, the affected area becomes more vulnerable to bacterial colonization and attachment loss [9]. Masters and Hoskins classified CEPs into three grades according to their proximity to the furcation entrance [10]. Grade III lesions have been associated with localized furcation involvement [6]. Enamel pearls (EPs) are ectopic nodules of enamel located near the CEJ or within the furcation. They share the same biological feature as CEPs and may further impair access for scaling and root planing [7,11].

The reported prevalence of these anomalies varied markedly across studies. Early skull-based investigations reported the prevalence of CEPs to be approximately 10% [12]. However, a recent cone-beam computed tomography (CBCT) study in a Korean population reported their prevalence to be as high as 76% [13]. Two CBCT studies from Turkey reported the prevalence of EPs ranging from 4.29% to 4.69%. Most EPs were detected in maxillary molars, especially the third molars and second molars [14,15]. The variability reflects both true ethnic differences and differences in detection methodologies.

Three-dimensional (3D) imaging consistently identifies more lesions compared to clinical examination or two-dimensional (2D) radiography. 2D radiography is limited by anatomical superimposition and restricted access to the cervical region. CBCT has therefore become the preferred modality for the in vivo assessment of these features [13].

To our knowledge, the prevalence of CEPs and EPs in a Saudi adult population has not been previously reported. Locally derived estimates are needed to inform clinical awareness and treatment planning. The aim of the present study was to determine the prevalence and distribution of CEPs and EPs in a sample of Saudi adults using CBCT.

## 2. Materials and Methods

### 2.1. Study Design and Ethics

This was a retrospective, cross-sectional, radiographic study designed to estimate the prevalence of cervical enamel projections and enamel pearls in maxillary and mandibular molars using CBCT. Ethical approval was obtained from the Institutional Review Board (NRR25/049/3) of King Abdullah International Medical Research Center (KAIMRC). Individual informed consent was waived given the retrospective design and the use of de-identified data. Patient confidentiality was maintained.

### 2.2. Setting, Population, and Sampling

The study was conducted at King Abdulaziz Medical City, Riyadh, in the Postgraduate Periodontics clinics. The source population comprised the 1376 patients who attended the postgraduate periodontics dental residents’ clinics at the National Guard Health Affairs between January 2024 and February 2025. Eligible records were identified using consecutive sampling. Every patient seen during this period was screened against the eligibility criteria, and all records meeting the criteria were included.

Patients aged 18 years or older with at least one diagnostic-quality CBCT scan that included the maxillary and/or mandibular first or second molars were eligible for inclusion. Patients were excluded if they had no CBCT scan on file or had lost all maxillary and mandibular first and second molars. CBCT scans of poor diagnostic quality were also excluded, including those with significant motion artefact, beam-hardening, or metallic artefacts obscuring the cervical region of the molars. Individual molars with extensive crown destruction, full-coverage metallic restorations, prior endodontic surgery, root amputation, or hemisection that precluded reliable evaluation of the cementoenamel junction were excluded.

### 2.3. Sample Size

The required sample size was calculated for the primary outcome, which is the prevalence of CEPs. The expected prevalence was set at 76%, based on the CBCT study of Lim et al. [13]. Using the standard formula for a single proportion, *n* = Z^2^ · *p* · (1 − *p*)/e^2^, with Z = 1.96, *p* = 0.76, and a margin of error e = 0.065, we then applied the finite population correction for the source population. The minimum required sample size was 150 patients.

### 2.4. Data Collection and Radiographic Assessment

All the included scans were obtained using a Planmeca ProMax 3D Plus unit (Planmeca Co., Helsinki, Finland) with parameters of 90 kV and 5–11 mA and a voxel size of 200 µm.

The following patient-level variables were recorded: age, gender, medical history (e.g., diabetes mellitus, hypertension, dyslipidemia, ischemic heart disease) categorized as healthy or medically compromised, and the number of first and second molars assessable on the CBCT scan.

**Figure 1:**
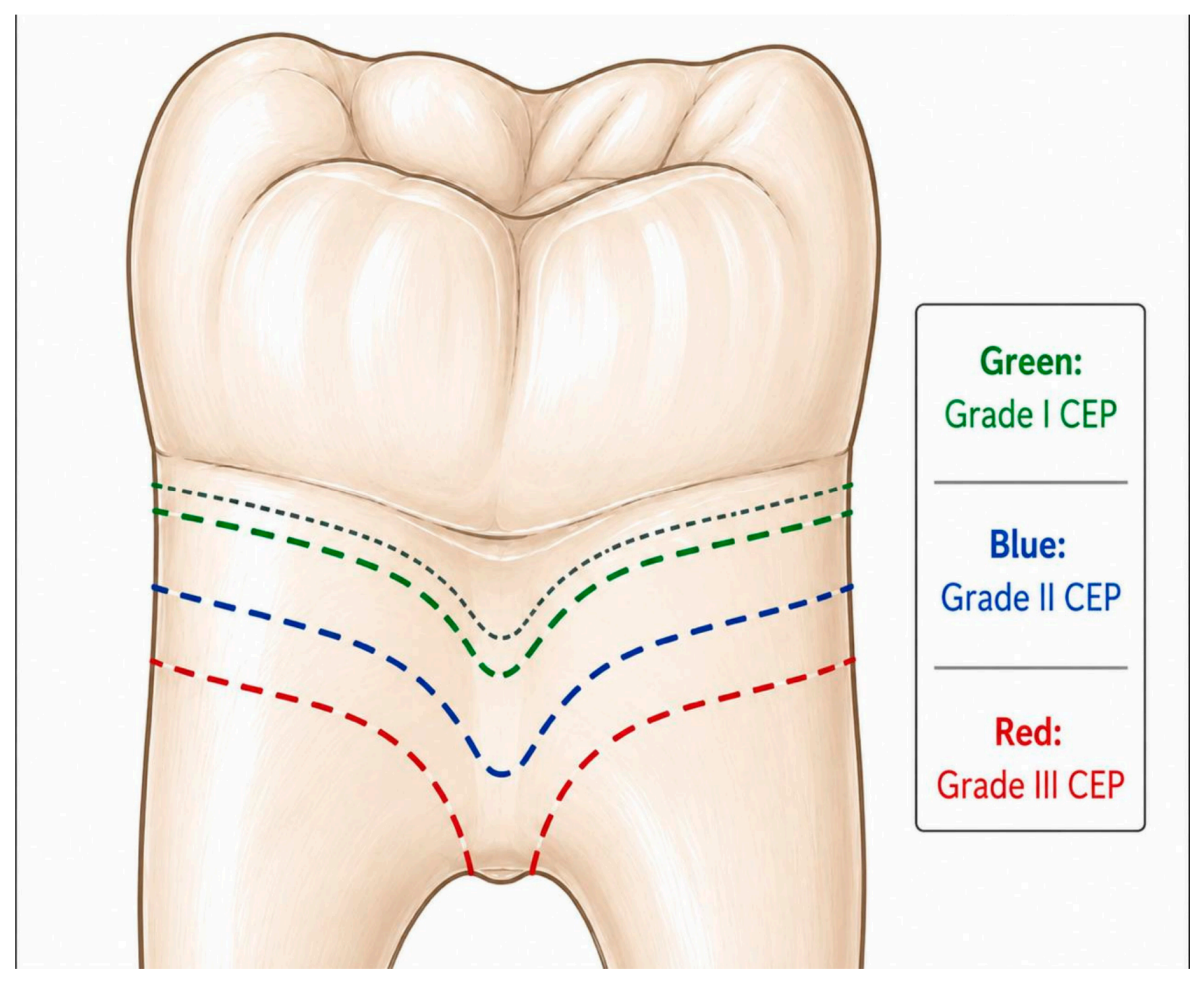
Classification of the cervical enamel projections.

At the tooth level, each molar was evaluated for the presence of a cervical enamel projection, defined as a continuous radiopaque enamel extension from the cementoenamel junction toward the furcation [13]. When present, CEPs were classified according to Masters and Hoskins [10] as Grade I, II, or III (Figure 1). The presence of an enamel pearl, defined as a well-defined radiopaque nodule at or near the cementoenamel junction [11], was also recorded. The affected tooth was identified as maxillary or mandibular and as first or second molar.

### 2.5. Examiner Calibration and Reliability

Three examiners (A.A., L.A., Y.A.) independently evaluated all CBCT scans after a calibration session using a set of training scans. Inter-rater reliability was assessed using Fleiss’s kappa coefficient. Kappa values were interpreted according to McHugh [16] as poor (<0.40), fair to good (0.40–0.75), or excellent (>0.75). Disagreements were resolved through discussion.

### 2.6. Statistical Analysis

Data were analyzed using RStudio (R 4.5.3, Posit Software, Boston, MA, USA). Continuous variables were summarized as mean ± standard deviation or median and interquartile range, depending on their distribution as assessed by the Shapiro–Wilk test. Categorical variables were summarized as frequencies and percentages. Prevalence estimates of CEPs and enamel pearls were reported at the patient and tooth levels, with 95% confidence intervals.

Bivariate associations with patient-level variables (age, gender, medical history) were examined using the chi-square test or Fisher’s exact test for categorical variables, and the independent-samples *t*-test or Mann–Whitney U test for continuous variables. The significance level was set at 5%.

## 3. Results

### 3.1. Study Characteristics

A total of 153 patients met the inclusion criteria during the study period (January 2024–February 2025), providing 732 maxillary and mandibular first and second molars assessable on CBCT. The mean age of the sample was 46.7 ± 15 years. Ninety-one patients (59.5%) were female and 62 (40.5%) were male. The number of assessable molars per patient ranged from 0 to 8 (median 5). Seventy-six patients (49.7%) had no reported systemic condition. Demographic characteristics are summarized in Table 1. Inter-rater agreement among the three investigators was excellent, with Fleiss’s κ = 0.86 for CEPs and κ = 1.00 for EPs.

### 3.2. Prevalence of CEPs

Cervical enamel projections were detected on CBCT in 45 of 153 patients, corresponding to a patient-level prevalence of 29.4% (95% CI 22.8–37.1%). At the tooth level, CEPs were identified on 56 of the 732 assessable molars, giving a tooth-level prevalence of 7.7% (95% CI 5.9–9.8%) (Table 2).

**Figure 2:**
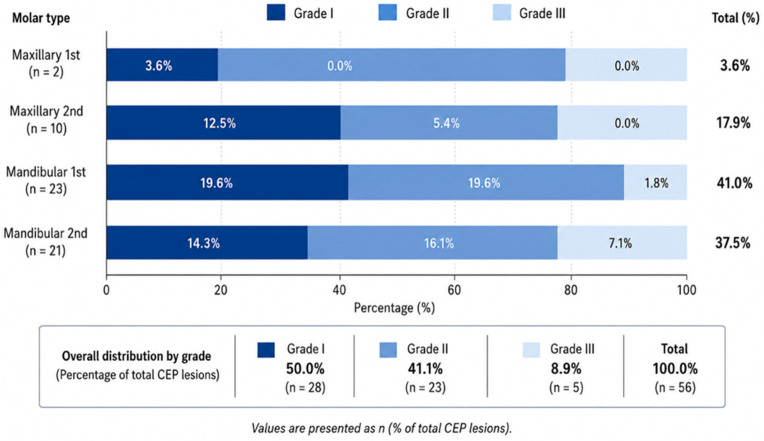
Distribution of Cervical Enamel Projections According to Grade and Affected Molar Type (Percentage of Total CEP Lesions, *N* = 56).

Among the 45 CEP-positive patients, the most frequent grade according to the Masters and Hoskins classification was Grade I (*n* = 28, 50.0%), followed by Grade II (*n* = 23, 41.1%) and Grade III (*n* = 5, 8.9%). Mandibular molars were the most commonly affected: mandibular first molars accounted for 41.1% of cases (*n* = 23) and mandibular second molars for a further 37.5% (*n* = 21). Maxillary second molars represented 17.9% (*n* = 10) and maxillary first molars only 3.6% (*n* = 2) (Figure 2).

### 3.3. Prevalence of EPs

**Figure 3:**
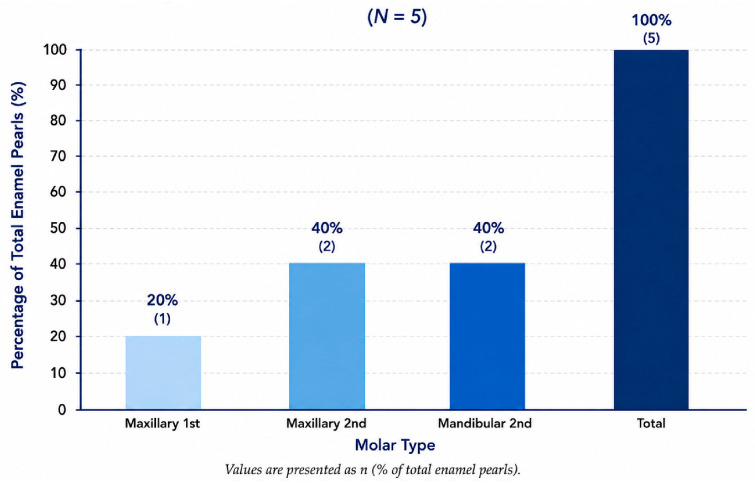
Anatomical Distribution of Enamel Pearls.

Enamel pearls were considerably less frequent than CEPs. Five of the 153 patients had at least one enamel pearl identified on CBCT, yielding a patient-level prevalence of 3.3% (95% CI 1.4–7.4%) and a tooth-level prevalence of 0.7% (5/732; 95% CI 0.3–1.6%) (Table 2). Among the five affected patients, the affected tooth was a maxillary second molar in two cases (40.0%), a mandibular second molar in two cases (40.0%), and a maxillary first molar in one case (20.0%) (Figure 3).

### 3.4. Exploratory Associations

No statistically significant associations were observed between the presence of CEPs and the patient-level variables examined. The prevalence of CEPs was 31.8% in females and 39.3% in males (χ^2^ test, *p* = 0.413). Median age was similar between CEP-positive (45 years, IQR 34–58) and CEP-negative patients (47 years, IQR 38–59; Mann–Whitney U test, *p* = 0.862). When patients were dichotomized into those with no reported systemic condition versus those with at least one condition, the prevalence of CEPs was 37.3% and 32.9%, respectively (χ^2^ test, *p* = 0.684).

Similarly, no statistically significant associations were observed for enamel pearls. The prevalence was 2.3% in females and 4.9% in males (Fisher’s exact test, *p* = 0.395), and 6.0% among patients with no reported systemic condition versus 1.2% among those with at least one condition (Fisher’s exact test, *p* = 0.209). Age did not differ significantly between EP-positive and EP-negative patients (Mann–Whitney U test, *p* = 0.094). Numerical results for all bivariate analyses are provided in Table 3.

## 4. Discussion

The present study reported the first CBCT-based assessment on the prevalence and anatomical distribution of CEPs and EPs in a Saudi adult population. The patient-level prevalence of CEPs was 29.4%. This is substantially lower compared to a Korean study reporting a prevalence of 76%, yet higher than results derived from a skull-based study in an Egyptian population [12,13]. This marked inter-study variability is well-documented in the literature and was recently reported to have a wide range (8.3–85.1%) [17]. The variability can be attributed to factors such as ethnicity and population-specific tooth morphology, which may exert a genuine biological influence on enamel extension patterns. Methodological heterogeneity also plays a role, including differences in detection modality, inclusion criteria, molar types evaluated, and anatomical surfaces examined. The detection modality itself contributes substantially to this variability.

Compared with panoramic and periapical radiography, CBCT eliminates anatomical superimposition and provides direct visualization of the buccal and lingual cervical surfaces, where CEPs most often occur. This typically yields higher prevalence estimates. However, CBCT voxel resolution (commonly 150–300 µm) is coarser than histological examination, and the smallest Grade I projections that approach the CEJ without a clear silhouette may be under-detected. Dry-skull studies, conversely, capture small lesions accurately but may exclude teeth lost during specimen preparation. These bidirectional biases partly explain why CBCT-based prevalence figures generally fall between those reported by histological and dry-skull studies.

Within CBCT itself, the choice of reconstruction view also influences detection. In the present study, the three-dimensional skull-reconstruction view was the most accurate for identifying CEPs and EPs, whereas the axial view alone was the least sensitive. This is consistent with the curved, vertical geometry of these lesions, which is best appreciated on rendered surface views rather than single-plane sections. Studies that rely primarily on axial or cross-sectional views may therefore underestimate prevalence relative to studies that incorporate three-dimensional reconstructions.

The present study found that CEPs are commonly found in mandibular first molars (41.1%), which reflects the unique anatomy of the bifurcation region in these teeth. The mandibular and maxillary teeth differ in furcation morphology, root trunk length, and interradicular anatomy, which may account for the relatively low occurrence of CEPs in maxillary first molars (3.6%). This finding is consistent with classic literature by Swan and Hurt in 1976 and further confirmed by recent papers [13,18]. Furthermore, the predominance of lower-grade CEPs (Grade I and II: 91.1% of lesions) in our study is consistent with the study reported by Lim in Koreans [13].

The low prevalence of enamel pearls in the present study (3.3% at the patient level; 0.7% at the tooth level) aligns closely with CBCT-based data from other populations. Zengin and colleagues (2022) reported a patient-level prevalence of 4.29% and a tooth-level prevalence of 0.71% in a Turkish cohort of 1003 CBCT scans [14].

The present study did not show a relationship between CEPs and gender. An earlier skull-based study reported a slight male predominance, while another CBCT study did not find a significant relationship [12,13]. CEPs are developmental anomalies that develop during tooth formation; they are unlikely to be acquired with advancing age or with medical conditions, which can explain the lack of correlation between age, medical history, and CEPs.

The findings of this study carry several points of significance. First, they provide the first CBCT-based prevalence estimates of CEPs and EPs in Saudi adults. Second, the patient-level CEP prevalence of 29.4% indicates that these lesions are common enough in routine periodontal practice to warrant systematic cervical-area review on any CBCT performed for molar assessment. Third, the predominance of mandibular first molars among CEP-affected teeth reinforces this region as a high-yield site for clinical screening, particularly in patients presenting with isolated furcation involvement of unclear aetiology.

Several clinical implications follow from these findings. When CBCT is acquired for periodontal or implant assessment, the cervical region of the molars should be examined routinely on three-dimensional reconstructions for CEPs and EPs, since both can be overlooked when only axial or panoramic-equivalent views are reviewed. Identification of a Grade II or III CEP on a molar with localized attachment loss should prompt consideration of odontoplasty as part of cause-related therapy. Detection of an enamel pearl before non-surgical periodontal treatment may also influence instrumentation strategy, since these lesions impede effective scaling and root planing. Finally, awareness of the local prevalence reported here may help clinicians counsel patients on the anatomical contribution to localized periodontitis when no other risk factor is evident.

Several limitations are found in the present study. First, the retrospective, single-center design limits the generalizability of the findings to the broader Saudi population. Because the sample was drawn exclusively from postgraduate periodontics clinics, it is likely enriched for patients with periodontal disease. As a result, it may not reflect the prevalence of these anomalies in the general Saudi adult population. The estimates reported here should therefore be interpreted as applying to a Saudi periodontal patient cohort. Secondly, while CBCT is the acknowledged reference standard for in vivo detection, its resolution is not equivalent to histological examination, and very small CEPs approaching the CEJ may have been missed. Thirdly, although CEPs—particularly Grade III lesions—have been linked to localized furcation involvement, the present study did not perform a direct tooth-level analysis correlating CEP presence or grade with concurrent furcation involvement on the same CBCT scans; this represents an important avenue for follow-up work. Fourth, the very small number of enamel-pearl-positive patients (*n* = 5) precluded meaningful subgroup analysis, and the absence of statistically significant associations for EPs should not be interpreted as evidence of no association. Finally, because the data were drawn from periodontics clinics, the number of missing or non-assessable molars was relatively high, which may have affected the observed prevalence estimates. As this was a retrospective study, the specific reasons for non-assessable molars (e.g., prior tooth loss versus exclusion due to restoration-related or imaging-related factors) were not systematically recorded and therefore could not be quantified separately. Future prospective, multicenter studies with standardized recording of tooth loss and reasons for tooth exclusion are needed to refine prevalence estimates in the Saudi population.

## 5. Conclusions

The patient-level prevalence of CEPs and EPs among Saudi adults, based on CBCT assessment, was 29.4% and 3.3%, respectively. Grade I CEPs were the most common, and most frequently affected mandibular first molars. Neither lesion was associated with age, gender, or medical history. This study highlights the value of CBCT in detecting these features. These dental anomalies may interfere with effective plaque control and periodontal instrumentation, potentially contributing to attachment loss.

## Figures and Tables

**Table 1:** Demographic characteristics of the study sample.

Characteristic	Value
Patients, *n*	153
Age, years, mean ± SD	46.7 ± 15
Female, *n* (%)	91 (59.5%)
Male, *n* (%)	62 (40.5%)
Healthy, *n* (%)	76 (49.7%)
Medically compromised, *n* (%)	77 (50.3%)
Total molars assessed, *n*	732
Molars assessed per patient, median (IQR)	5 (3–7)

**Table 2:** Prevalence of cervical enamel projections and enamel pearls at the patient and tooth levels.

Outcome	*n*/*N*	Prevalence (%)	95% CI
CEPs, patient-level	45/153	29.4	22.8–37.1
CEPs, tooth-level	56/732	7.7	5.9–9.8
EPs, patient-level	5/153	3.3	1.4–7.4
EPs, tooth-level	5/732	0.7	0.3–1.6

CI, confidence interval; CEPs, cervical enamel projections; EPs, Enamel pearl.

**Table 3:** Bivariate associations of cervical enamel projections and enamel pearls with patient-level variables.

Variable	CEPs (Test)	CEPs (*p*-Value)	EPs (Test)	EPs (*p*-Value)
Gender	Chi-square	0.413	Fisher exact	0.395
Age	Mann–Whitney U	0.862	Mann–Whitney U	0.094
Medical history	Chi-square	0.684	Fisher exact	0.209

*p*-values were calculated using chi-square, Fisher’s exact, or Mann–Whitney U tests as appropriate.

## Data Availability

The data presented in this study are available on request from the corresponding author. The data are not publicly available due to privacy restrictions.

## Abbreviations

The following abbreviations are used in this manuscript:

CBCT	Cone-beam computed tomography
CEJ	Cementoenamel junction
CEP	Cervical enamel projection
CI	Confidence interval
EP	Enamel pearl
IQR	Interquartile range
KAIMRC	King Abdullah International Medical Research Center
SD	Standard deviation
